# Surgical Resection for Small Cell Lung Cancer: Pneumonectomy versus Lobectomy

**DOI:** 10.5402/2012/101024

**Published:** 2012-05-30

**Authors:** Jiang Yuequan, Zhang Zhi, Xie Chenmin

**Affiliations:** Department of Thoracic Surgery, Chongqing Cancer Institute, Shapingba District, Hanyu Avenue, Chongqing 400030, China

## Abstract

*Background*. There are some patients with SCLC that are diagnosed in the operating room by cryosection and surgeons had to perform surgical resection for these patients. The aim of this study is to compare the effective of pneumonectomy with lobectomy for SCLC. *Methods*. A retrospective study was undertaken in 75 patients with SCLC that were diagnosed by cryosection during surgery. 31 of them underwent pneumonectomy, 44 underwent lobectomy. Local recurrence rate and survival rate according to surgical procedures and cancer stages were analyzed. *Results*. There was significant difference in the overall survival rate between lobectomy and pneumonectomy groups (*P* = 0.044). For patients with stage II SCLC, the overall survival rate after pneumonectomy was significantly better than after lobectomy (*P* = 0.028). No significant difference in overall survival rate was found between the two surgical groups in patients with stage III SCLC (*P* = 0.933). The local recurrence rate in lobectomy group was significant higher that in pneumonectomy group (*P* = 0.0017). *Conclusions*. SCLC was responsive to surgical therapy. When surgeons have to select an appropriate method of operation for patients with SCLC during surgery, pneumonectomy may be the right choice for these patients. Pneumonectomy can result in significantly better local control and higher survival rate compare with lobectomy.

## 1. Background

According to World Health Organization (WHO) statistics, more than 1 million cases of lung cancer are diagnosed annually around the world. The incidence of small cell lung cancer (SCLC) was about 20–25% of all newly diagnosed lung cancers [1]. SCLC is considered distinct from other lung cancers, because of their clinical and biologic characteristics. It exhibits aggressive behavior, with rapid growth and early spread. SCLC seems sensitive to both chemotherapy and radiotherapy, but the overall 5-year survival rate is still poor despite the sensitivity [2]. Although the efficacy of surgery for SCLC is controversial, surgical excision is still believed a curative treatment. In fact, some patients with SCLC were diagnosed in the operating room by cryosection. For these patients the surgeon had to choose proper surgical procedure. We found some patients with SCLC who underwent pneumonectomy experienced long-term survival. We supposed that pneumonectomy might achieve complete resection and conferred a survival advantage for these patients. This study reviewed the records of 75 patients with SCLC diagnosed by intraoperative cryosection and compared the therapeutic efficacy of pneumonectomy and lobectomy on patients with SCLC.

## 2. Methods

From January 1982 to December 2010, there were 85 patients did not that a confirmed diagnosis of SCLC before resection and underwent surgery at the Department of Thoracic Surgery of Chongqing Cancer Hospital & Institute. For 51 of the 85 patients (60%), histological or cytological diagnosis was not obtained preoperatively. For the remaining 34 patients, the preoperative diagnosis of adenocarcinoma was in 11 cases, bronchioloalveolar carcinoma in 11 cases, squamous cell carcinoma in 12 cases.

2 patients had an incomplete resection, and 1 patient had unresectable disease. 6 patients had the pathologic subtype with combined histology tumor (mixtures of SCLC with non-SCLC components). 1 patient died of perioperative complications. Thus 75 patients with SCLC were in this study. This study was approved by the Ethics Committee of Chongqing Cancer Hospital & Institute, China.

There were 69 men and 6 women, with the median age of 56 years (range 41–71 years). The preoperative assessments included chest roentgenography, computed tomography of the chest, external ultrasonography of the abdomen and bone scintigraphy. Magnetic resonance imaging of the brain was used in 51 patients. In this study, 61 patients underwent bronchoscopy and 23 patients underwent mediastinoscopy without definite diagnosis of SCLC. 56 patients get PET-CT (Positron emission tomography-computed tomography). Because all the patients in this study had no pathological diagnosis of SCLC preoperatively, induction chemotherapy was not performed.

All operations were performed with curative intent and every patient underwent mediastinal lymph node resection. Pathologic staging was undertaken according to the 7th edition of the AJCC staging system of lung cancer. All these patients were referred for consideration of adjuvant chemotherapy and prophylactic cranial irradiation (PCI). The postoperative chemotherapy was performed with the PE regimen that is etoposide and either cisplatin or carboplatin. Four to six cycles of chemotherapy were performed if the patient's condition after surgery was well tolerable against the treatment. 8 patients were not treated with PCI and 5 of the 8 patients were not treated with adjuvant chemotherapy.

### 2.1. Followup

Following hospital discharge, patients with SCLC were regularly monitored in the outpatient department at intervals of 1 month for the first 1 year, 3 months for the next 2 years, and every 6 months thereafter. All patients in this study underwent a clinical evaluation that included chest radiography, external ultrasonography of the abdomen, computed tomography (CT) scans of the thorax, and bone emission computed tomography (ECT) scanning at least once half year. Local recurrence was defined as recurrence that occurred within the ipsilateral hemithorax including the mediastinum.

### 2.2. Statistical Analysis

Survival was defined as the interval between date of surgery and date of death or last followup. Survival rates were calculated using the Kaplan-Meier method and the differences were compared using the log-rank test. Comparisons of continuous and dichotomous variables between groups were performed with the Student *t*-test and *χ* ^2^ tests, respectively. All analyses were accomplished with SPSS 13 statistical package.

## 3. Results

### 3.1. Surgery

31 patients underwent pneumonectomy (including 7 right and 24 left pneumonectomies). 44 patients underwent lobectomy (including 12 patients underwent sleeve resection). The lobectomy procedures included 12 right upper lobectomies, 1 middle lobectomy, 2 right upper and middle lobectomies, 2 middle and right lower lobectomies, 9 right lower lobectomies, 10 left upper lobectomies, and 8 left lower lobectomies. The patients were divided into two: groups pneumonectomy group (*n* = 31) and lobectomy group (*n* = 44).

### 3.2. Characteristics of the Patients

The clinical and pathologic characteristics of the two group patients are presented in Table 1. There were no statistical differences between the two groups regarding age, sex, and adjuvant therapy. The pathologic stage of the lobectomy group was stage I in 2, stage II in 29, and stage III in 13 patients. The pathologic stage of pneumonectomy group was stage II in 24 and stage III in 7 patients. There were no patients with stage I in pneumonectomy group. Statistic analysis showed that there was no significant difference in distribution of pathologic stage between the two groups (*P* = 0.249).

### 3.3. Survival Rate of the Patients and Local Recurrence with SCLC

**Figure 1:**
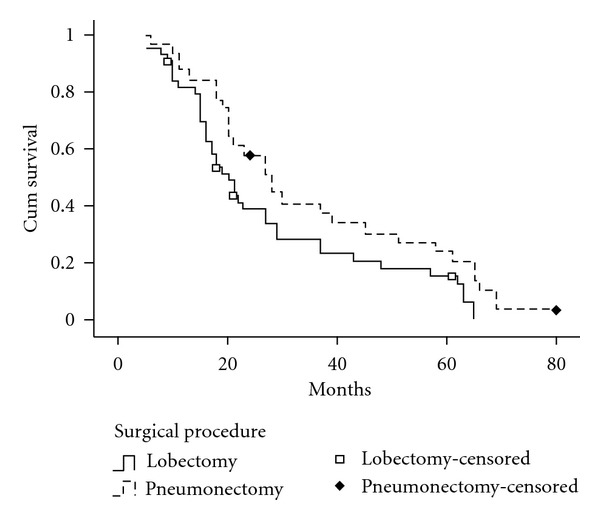
Survival curves according to surgical procedures. The 5-year survival rate for patients with SCLC was 16.1% by lobectomy, 24.0% by pneumonectomy. There was significant difference in the overall survival rate between the two groups (*P* = 0.044).

The median survival time and 5-year survival rate for entire cohort were 22 months and 20.34%. They were 27 months and 26.2% for stage II, 18 months and 0.0% for stage III. The median survival time and 5-year survival rate of patients with SCLC were 20 months and 11.1% by lobectomy, 28 months and 24.0% by pneumonectomy (Table 2). There was significant difference in the overall survival rate between the two surgical groups (*P* = 0.044; Figure 1).

**Figure 2:**
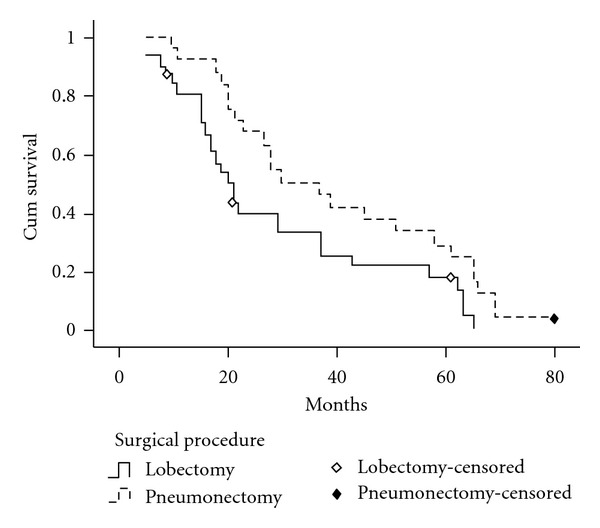
Survival curves of patients with stage II SCLC according to surgical procedures. The 5-year survival rate of patients with stage II SCLC was 16.7% in lobectomy group, 31.6% in the pneumonectomy group. For patients with stage II SCLC, the overall survival rate after pneumonectomy was significantly better than after lobectomy (*P* = 0.028).

**Figure 3:**
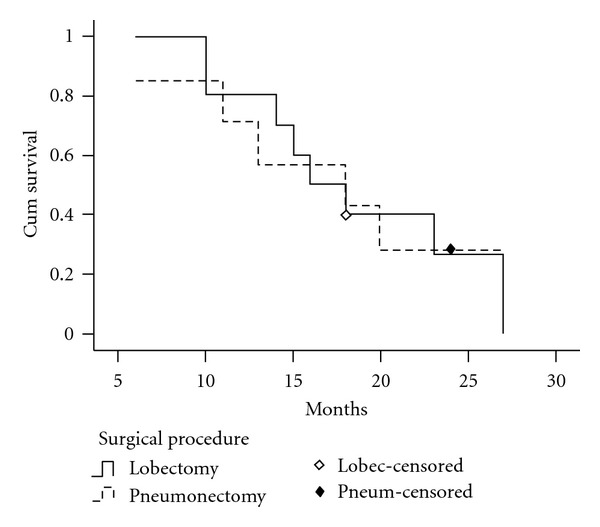
Survival curves of patients with stag III SCLC according to surgical procedures. No significant difference in overall survival rate was found between lobectomy group and pneumonectomy group in patients with stage III SCLC.

The median survival time and 5-year survival rate of patients with stage II SCLC were 22 months and 16.7% in lobectomy group, 30 months and 31.6% in the pneumonectomy group. For patients with stage II SCLC, the overall survival rate after pneumonectomy was significantly better than after lobectomy (*P* = 0.028, Figure 2). For patients with stage III SCLC, the median survival time was 16 months in lobectomy group and 18 months in pneumonectomy group respectively. There was no patient with stage III SCLC who survived for more than 5 years in this study. No significant difference in overall survival rate was found between lobectomy group and pneumonectomy group in patients with stage III SCLC (*P* = 0.933, Figure 3).

The local recurrence rate comparison of two surgical procedures was showed in Table 2. The local recurrence rate were 59.1% (26/44) in lobectomy group, 22.6% (7/31) in pneumonectomy group, there was statistically significant difference in local recurrence rate between the two groups (*P* = 0.0017). By stages, there was statistically significant difference of local recurrence rate between the two surgical groups in stage II SCLC (*P* = 0.002), but no significant difference of local recurrence rate was found between the two groups in stage III SCLC (*P* = 0.3348).

**Figure 4:**
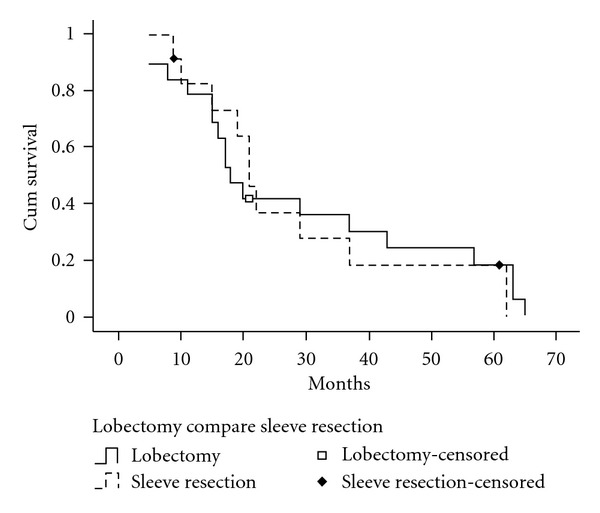
Survival curves of patients according to surgery of sleeve resection lobectomy and lobectomy. There was no significant difference in overall survival rate between the patients who underwent general lobectomy and these who underwent sleeve resection lobectomy (*P* = 0.877).

In our study, the patients with sleeve resection were included into lobectomy group, because there was no significant difference in overall survival rate between the patients who underwent general lobectomy and these who underwent sleeve resection lobectomy (*P* = 0.877, Figure 4). 

## 4. Discussion

The efficacy of surgery in SCLC is controversial. About 30 years ago, British Medical Research Council performed a randomized trial about surgery versus radiotherapy for SCLC. The result showed that surgery and radiotherapy were equally ineffective in limited stage SCLC [2–4]. This result has been widely cited as evidence to prove that surgical treatment to SCLC is ineffective. But proponents of surgery argue that there were some limitations in that randomized trial. CT scanning and mediastinoscopy were unavailable at that time. The patients recruited in that trial were not currently suitable for surgery, complete resection was only achieved in 34 (48%) patients and 37 (52%) patients underwent exploratory thoracotomy only.

With the advent of new diagnostic tools, such as spiral computed tomography and positron emission tomography, limited disease can be more readily identified and adequately staged preoperatively. Some clinicians believed that good results can be achieved in selected patients with complete resection [5, 6]. Moreover, with the platinum agent, granulocyte-colony-stimulating factor, and serotonin-antagonizing antiemetic agent becoming available, the chemotherapeutic regimens for SCLC have been changed [7–10]. Recent studies reported that multimodality treatment involving surgery achieved a good prognosis in SCLC patients with limited stage disease, thus suggested the importance of surgery with a curative intent [11, 12].

Although many researches confirmed that multimodality treatment including surgery and chemotherapy might represent an effective form of treatment for limited SCLC, it was generally accepted that surgical resection was appropriate only for the patients with stage I SCLC [13, 14]. However, stage of SCLC is usually underestimated preoperatively [2, 15]. Lymph node metastasis is often underestimated, and occult mediastinal involvement might be missed even by medistinoscopy. Eric Lim and his colleagues reported 59 patients with stage I to III SCLC who underwent lung resection with nodal dissection and showed excellent overall 5-year survival of 52%. Their surgical series suggests that good results can be achieved in selected patients with complete resection throughout the spectrum of UICC stage I to III [5].

Our study reviewed 75 patients that do not have confirmed diagnosis of SCLC preoperatively. The reason is that there were no CT and bronchoscope in our institute until 1989. So, not every patient in this group gets these assessments even after 1989 because of economic reason. Also some of these patients were misdiagnosed as NSCLC. Their postoperative pathologic stages included stage I in 3 cases, stage II in 55 cases, and stage III in 17 cases. The median survival time and postoperative 5-year survival rate of patients with SCLC in our surgical series were 22 months and 20.34%. These figures can be compared with the results reported by Brock et al. [14] and are poorer than the result reported by Inoue et al. [15].

Comparing the median survival time and survival rate of two surgical procedures, we found that the median survival time and 5-year survival rate by pneumonectomy were better than those by lobectomy (28 months, 24.0% versus 20 months, 11.1%), and statistical analysis showed there was significant difference in overall survival rate between the two groups (**P** = 0.044). Moreover, our study showed that for patients with stage II SCLC, the postoperative overall survival rates by pneumonectomy were significantly better than by lobectomy (*P* = 0.028). For patients with stage III SCLC, there was no difference between the two surgical groups in overall survival rates (*P* = 0.933).

We had examined 31 cases of pneumonectomy for SCLC in this study. It was because we found that SCLC usually originates in the lung's large central airways, invades the main bronchus and fuses with metastatic hilar lymph nodes at presentation. The metastatic lymph nodes usually involve interlobular lymph node and peribronchial lymph nodes of neighboring lobe also. Lobectomy often leaves these peribronchial lymph nodes in neighboring lobe. It is even impossible to distinguish primary tumor from lymph node metastasis during operation. Shepherd reported that local control remains a problem, with one-third of patients having recurrence only at the primary site. Failure to achieve control at the primary site remains the single most important obstacle to cure in patients with limited SCLC [16]. We believe that pneumonectomy can achieve complete resection of the neoplasm for SCLC rather than lobectomy do. In this study, the local recurrence rate of patients with SCLC was 59.1% (26/44) in lobectomy group, and it was 22.6% (7/31) in pneumonectomy group. There was significant difference between the two groups in local recurrence rate (*P* = 0.0017). The results indicate that pneumonectomy can afford better curability rates than lobectomy.

## 5. Conclusion

Chemotherapy in combination with radiation therapy is the mainstay of treatment for SCLC. But there are still some patients with SCLC that are diagnosed intraoperatively. In this specific situation, the surgeon must select an appropriate method of operation to complete the operation for these patients. In our study, we can easily draw the conclusion that pneumonectomy can achieve complete resection and reduce local recurrence rate of patients with SCLC. It can result in better local control than lobectomy. The survival of patients with SCLC after pneumonectomy is better than after lobectomy.

Nevertheless, there were some limitations in this study which should be noted. The patients in the two surgical groups were not selected by randomization. In pneumonectomy group, 70 years old was the upper limit age of patients. Patients with pneumonectomy had healthy lung function and strong heart. These factors may influence on survival rate. Also the long-term complications of the two surgical procedures were not analyzed in this study. We had mediastinoscopy instrument after 2000: only 23 patients in this study received mediastinoscopy. In recent 15 years, we seldom performed surgery to patients with SCLC, because we also think that surgical intervention in the management of SCLC is not considered standard. So most of the cases in this study were before 15 years ago.

## Figures and Tables

**Table 1:** Characteristics of the patients with lobectomy and pneumonectomy.

Characteristic	Lobectomy (*n* = 44)	Pneumonectomy (*n* = 31)	*P* value
Age mean ± SD (years)	58.5 ± 18.4	57.9 ± 17.8	0.8609
Gender			
Male	40	29	0.6782
Female	4	2	
Adjuvant therapy			
Chemotherapy alone	1	2	0.3631
Chemotherapy plus PCI	43	29	
Pathologic stage			
I	3	0	
II	31	24	0.3272
III	10	7	

**Table 2:** Comparison of Local recurrence and survival rate.

	Lobectomy	Pneumonectomy	*P*
All stage			
Median survival time (CI)	20 (15.83–24.16) *n* = 44	28 (21.52–34.48) *n* = 31	
5-year survival rate	11.1%	24.0%	0.044
Local recurrence rate	59.1% (26/44)	22.6% (7/31)	0.0017
Stage II			
Median survival time (CI)	21 (17.03–24.97) *n* = 31	30 (18.48–41.52) *n* = 24	
5-year survival rate	16.7%	31.6%	0.028
Local recurrence rate	61.3% (19/31)	20.8% (5/24)	0.0027
Stage III			
Median survival time (CI)	16 (11.35–20.65) *n* = 10	18 (5.16–30.83) *n* = 7	
5-year survival rate	0	0	0.933
Local recurrence rate	60% (6/10)	28.6% (2/7)	0.3348

CI: 95% confidence interval.
